# Supplementary material for: Integrating Wikipedia editing into health professions education: a curricular inventory and review of the literature
Source: Perspect Med Educ. 2020 Oct 8;9(6):333–42. doi: 10.1007/s40037-020-00620-1 (PMC7718341; doi:10.1007/s40037-020-00620-1)
Supplement: Supplementary file 1 — Appendices A and B [file 40037_2020_620_MOESM1_ESM.docx]

**Appendix A: Search Strategies**

**OVID Medline**

Wikipedia.af.

Education, Professional.sh

Students, Health Occupations.sh

Schools, Health Occupations.sh

“medical education”.ab,ti.

"medical student*".ab,ti.

“nursing student*”.ab,ti.

“dental student*”.ab,ti.

“pharmacy student*”.ab,ti.

“veterinary student*”.ab,ti.

“medical school*”.ab,ti.

“nursing school*”.ab,ti.

“veterinary school*”.ab,ti.

“pharmacy education”.ab,ti.

“nursing education”.ab,ti.

“health profession* student*”.ab,ti.

2 or 3 or 4 or 5 or 6 or 7 or 8 or 9 or 10 or 11 or 12 or 13 or 14 or 15 or 16

English.lg

1 AND 17 AND 18

**Embase**

('wikipedia'/exp OR wikipedia) AND ('health student'/exp OR 'curriculum'/exp OR 'medical education'/exp OR 'paramedical education'/exp OR curriculum OR ((medical OR nursing OR pharmacy OR dental OR veterinary OR “health professional”) NEXT/2 (student* OR education OR school*)):ab,ti) AND [english]/lim

**ERIC**

wikipedia AND (healthcare OR "health care" OR "health professional" OR medical OR medicine OR nursing OR dental OR veterinary OR pharmacy)

**Web of Science**

wikipedia AND (healthcare OR "health care" OR "health professional*" OR medical OR medicine OR nurs* OR dent* OR veterinar* OR pharm*) AND (student* OR education OR school* OR curriculum)

**Appendix B: WikiEdu modules included on course dashboards (# of dashboards; %)**

| Wikipedia policies (27; 100%) |
| --- |
| Evaluating articles and sources (25; 93%) |
| Adding citations (25; 93%) |
| Contributing images and media files (25; 93%) |
| Editing health and psychology topics (25; 93%) |
| Plagiarism and copyright violation (24; 89%) |
| Peer review (21; 78%) |
| How to edit: Wikicode vs Visual Editor (20;74%) |
| Sandboxes, talk pages, and watchlists (20; 74%) |
| Continue improving your article (14; 52%) |
| Drafting in the sandbox (14; 52%) |
| Evaluate Wikipedia (14; 52%) |
| Finalizing your topic and finding sources (13; 48%) |
| Finding your article (12; 44%) |
| Moving work out of the sandbox (12; 44%) |
| Add to an article (12; 44%) |
| What’s a content gap? (10; 37%) |
| Choose your article (10; 37%) |
| Thinking about sources and plagiarism (10; 37%) |
| Thinking about Wikipedia (9; 33%) |
| Editing basics (7; 26%) |
| Moving work out of the sandbox (as a group) (6; 22%) |
| In-class presentation (6; 22%) |
| Sandboxes and Mainspace (5; 19%) |
| Did you know? *Students nominate their article the DYK? section of Wikipedia’s Main Page - DYK? features short facts about recently edited articles.* (4; 15%) |
| Drafting in the sandbox (as a group) (3; 11%) |
| Assigning medical topics (3;11%) |
| Copyedit an article (3; 11%) |
| Reflective essay (3; 11%) |
| Designing a Wikipedia writing and research assignment (1; 4%) |
| Finding articles (for instructors) (1; 4%) |
| Translating articles (1; 4%) |

| **Wikipedia Topic Guides that were featured in courses:**  Medicine (13), Psychology (6), Science Communication (6), LGBT+ Studies (3), Environmental Science (2), Genes and Proteins (2), Women’s Studies (1), Political Science (1), Books (1), Chemistry (1), History (1) |
| --- |
