# Supplementary figures and images for: Integrating Wikipedia editing into health professions education: a curricular inventory and review of the literature
Source: Perspect Med Educ. 2020 Oct 8;9(6):333–42. doi: 10.1007/s40037-020-00620-1 (PMC7718341; doi:10.1007/s40037-020-00620-1)

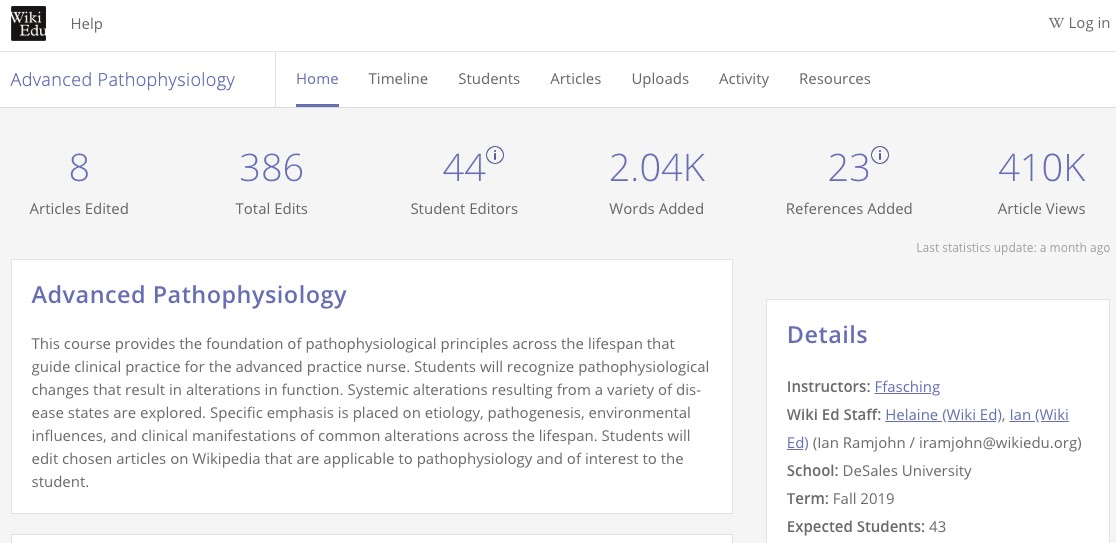

Supplement: Supplementary file 2 — Fig. 1: A screenshot of the Wiki Edu dashboard for the course Advanced Pathophysiology offered to 44 advanced practice nurses at DeSales University in Fall 2019 [30] [file 40037_2020_620_MOESM2_ESM.jpg]

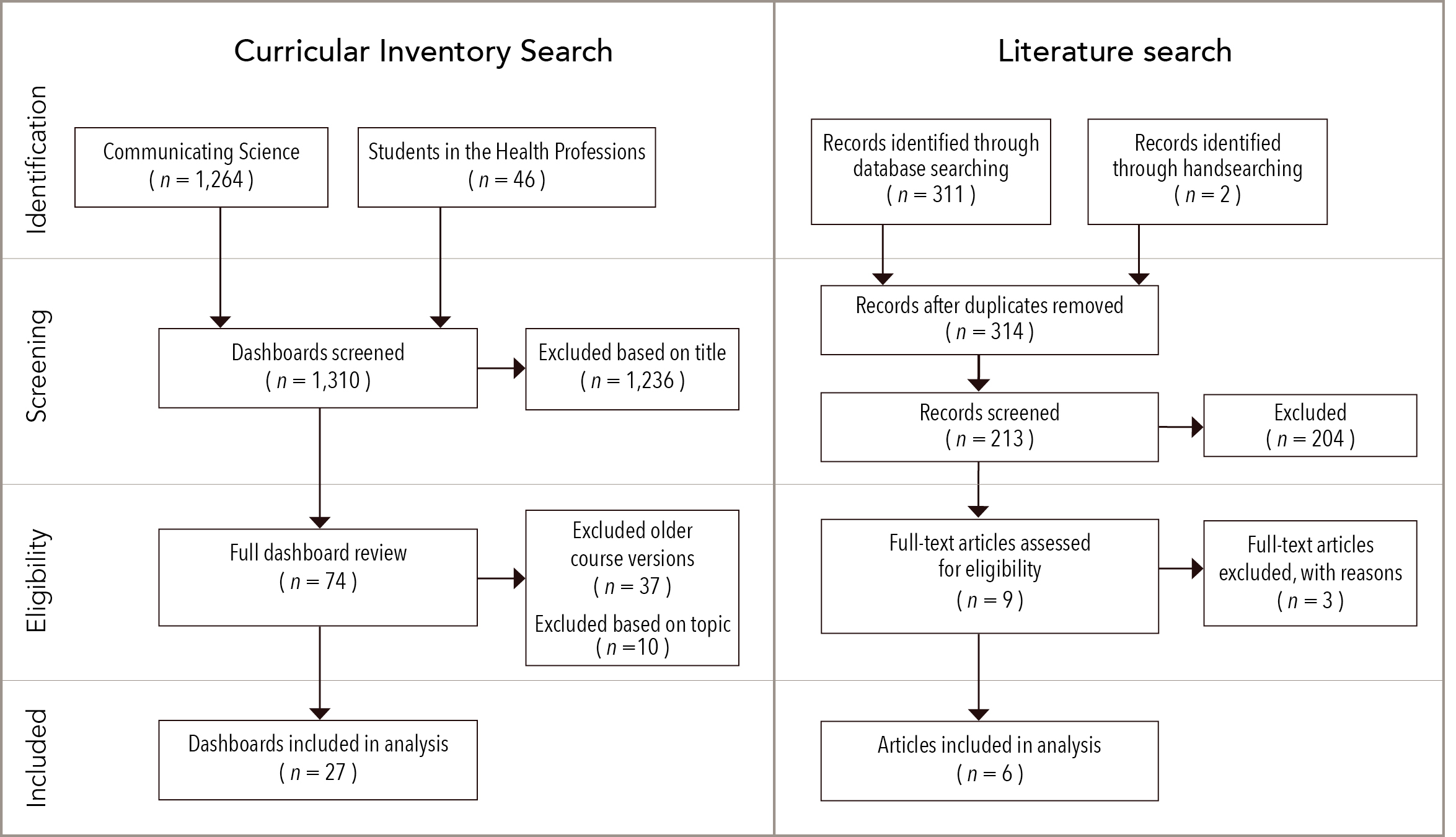

Supplement: Supplementary file 3 — Fig. 2: The search and selection process for curricular materials and literature search that describe educational interventions for health professions students that use Wikipedia [file 40037_2020_620_MOESM3_ESM.jpg]
