# Supplementary material for: Integrating Wikipedia editing into health professions education: a curricular inventory and review of the literature
Source: Perspect Med Educ. 2020 Oct 8;9(6):333–42. doi: 10.1007/s40037-020-00620-1 (PMC7718341; doi:10.1007/s40037-020-00620-1)
Supplement: Supplementary file 4 — Table: Course objectives provided by instructors in WikiEdu dashboards for teaching Wikipedia editing to HPE students [file 40037_2020_620_MOESM4_ESM.docx]

Course objectives provided by instructors in WikiEdu Dashboard for teaching Wikipedia editing to HPE students.

| Author | Course objectives |
| --- | --- |
| Apollonio, 2018 [12] | * Develop the professional skills associated with pharmacy practice by improving medicines information in Wikipedia. |
| Azzam, 2017 [11] | * Hone information retrieval and assessment skills;  * Practice communicating medical knowledge to an exceptionally broad global audience;  * Expand their sense of the health provider’s roles in the Internet Age. |
| Badgett, 2011 [14] | * Learn components of evidence-based practice. |
| Evenstein, 2017 [49] | * Create quality medical-related content in Wikipedia;  * Be better consumers of online information;  * Become active members of the information culture by taking part in a collaborative construction of knowledge. |
| Murray, 2020 [13] | * Evaluate Wikipedia as a platform for sharing medical information;  * Search for and locate medical information;  * Enhance Wikipedia using EBM skills;  * Analyze claims of a Wikipedia article on complementary and alternative medicine. |
